# *PADI4* rs2240337 G>A polymorphism is associated with susceptibility of esophageal squamous cell carcinoma in a Chinese population

**DOI:** 10.18632/oncotarget.20675

**Published:** 2017-09-06

**Authors:** Liming Wang, Haiyong Gu, Tao Long, Huiwen Pan, Lu Lv, Yijun Shi, Jingfeng Zhu, Yangyong Sun, Weifeng Tang, Guowen Ding, Suocheng Chen, Yu Fan, Hao Ding, Cheng Qian, Qun Wang, Jun Yao, Lijie Tan, Jun Yin

**Affiliations:** ^1^ Cancer institute, Department of Chemotherapy, Affiliated People’s Hospital of Jiangsu University, Zhenjiang, Jiangsu, China; ^2^ Department of Cardiothoracic Surgery, Affiliated People’s Hospital of Jiangsu University, Zhenjiang, Jiangsu, China; ^3^ Department of Respirology, Affiliated People’s Hospital of Jiangsu University, Zhenjiang, Jiangsu, China; ^4^ Department of Thoracic Surgery, Zhongshan Hospital of Fudan University, Shanghai, China; ^5^ Department of Gastroenterology, Affiliated People’s Hospital of Jiangsu University, Zhenjiang, Jiangsu, China

**Keywords:** PADI4, polymorphisms, esophageal squamous cell carcinoma, molecular epidemiology

## Abstract

**Background:**

Esophageal cancer (EC) remains one of the major causes of cancer incidence and mortality worldwide. Genetic factors, such as single nucleotide polymorphisms (SNPs), may contribute to the carcinogenesis of EC.

**Methods:**

We conducted a hospital based case-control study to evaluate the genetic susceptibility of SNPs on the development of EC. A total of 629 esophageal squamous cell carcinoma (ESCC) cases and 686 controls were enrolled for this study. Seven *PADI4* SNPs were determined by ligation detection reaction method.

**Results:**

Our findings suggested that the *PADI4* rs2240337 GA/AA variants were significantly associated with decreased risk of ESCC. Haplotype *PADI4* A_rs2477137_C_rs1886302_G_rs11203366_G_rs16825533_G_rs2240337_A_rs1635564_A_rs1635562_ and C_rs2477137_T_rs1886302_G_rs11203366_A_rs1635564_G_rs2240337_C_rs1635564_T_rs1635562_ polymorphism was correlated with decreased susceptibility to ESCC, while C_rs2477137_T_rs1886302_A_rs11203366_A_rs1635564_G_rs2240337_A_rs1635564_A_rs1635562_ was correlated with increased susceptibility of ESCC. Stratification analyses demonstrated that smoking significantly increased ESCC risk in *PADI4* rs11203366 AG/AA, rs1886302 CC/CT, rs1635562 AT, rs1635564 CA and rs2477137 AC genotype. Alcohol drinking increased ESCC risk in *PADI4* rs11203366 AG, rs1635562 AT, rs1635564 CA, rs2477137 AC, rs1886302 CT genotype. In younger cohort (<63 years), rs11203366 AA genotype was associated with increased risk of ESCC. *PADI4* rs1886302 CC variant was associated with ESCC susceptibility in female cohort.

**Conclusions:**

Our study suggested that *PADI4* rs2240337 G>A polymorphism may be correlated with individual susceptibility to ESCC. *PADI4* rs11203366, rs1886302, rs1635562, rs1635564 and rs2477137 polymorphisms were implicated with altered susceptibility of ESCC based on sex, age, smoking status and alcohol consumption. However, larger studies among different ethnic populations and further experiments using genetically mutated cells or animals are warranted to verify our conclusion.

## INTRODUCTION

Esophageal cancer is one of the most common cancers worldwide, and carries a high mortality after diagnosis following the onset of symptoms [1]. Cancer of the esophagus occurs in two major histological forms, esophageal squamous cell carcinoma (ESCC) and esophageal adenocarcinoma (EAC). ESCC dominates in most parts of the world, especially in high-risk areas such as China, where it accounts for about 90% of the total esophageal cancer cases [2, 3]. Smoking and alcohol consumption are related with more than 90% of ESCC patients in the western countries [4, 5], but the role of smoking and alcohol consumption is less important in China. The risk factors for ESCC in China include poor nutrition, lack of fruit and vegetables, drinking hot beverages and opium [3, 6].

The peptidylarginine deiminase IV (PADI4 or PAD4) converts arginine residues at histone tails to citrulline [7]. PADI4 has been demonstrated to co-localize with cytokeratin, an intermediate filament protein that plays a role during cell differentiation and apoptosis [8–10]. In cancer, high PADI4 expression has been connected to tumor growth [11], as PADI4 was overexpressed in numerous malignant cancers, but not in healthy tissues [8]. Recent study using immunohistochemistry further verified a significant PADI4 expression in various malignancies, comprising esophageal squamous cancer cells [12]. Consistently, PADI4 level in the blood increased dramatically in the patients with various malignant tumors, but considerably declined after tumor excision surgery [12]. Notably, PADI4 can disrupt the apoptotic process via the citrullination of histone H3 in the promoter of p53-target genes [13]. Therefore, we postulated that PADI4 might play an important role in the carcinogenesis of the esophageal cancer.

Single nucleotide polymorphisms (SNPs) account for more than 90% genetic variations. Despite the evidence described above indicated a correlation between *PADI4* and ESCC, few molecular epidemiological studies have explored the relationship between *PADI4* SNPs and susceptibility of ESCC with inconsistent results [13]. In a small cohort of esophageal cancer patients (including ESCC and EAC), *PADI4* rs10437048 and rs41265997 were found significantly associated with the risk of esophageal cancer [13]. To specifically examine the potential associations between genetic variants in *PADI4* and ESCC risk, we studied the correlation with the tagging SNP strategy in a larger cohort of 629 subjects of ESCC and 686 controls.

## RESULTS

### Characteristics of the study population

Characteristics of cases and controls included in the study are summarized in Table 1. The cases and controls appeared to be adequately matched on age and sex as suggested by the *χ*^2^ test. As shown in Table 1, significant difference was detected on smoking status (*p*<0.001) between the cases and the controls, and drinking rate (*p*<0.001) was higher in ESCC patients than in control subjects.

**Table 1:** Distribution of selected demographic variables and risk factors in ESCC cases and controls

Variable	Cases (n=629)		Controls (n=686)		*p* ^a^
	n	%	n	%	
**Age (years)** mean ± SD	62.85 (±8.13)		62.58 (±7.89)		0.541
**Age (years)**					0.155
< 63	310	49.28	365	53.21	
≥ 63	319	50.72	321	46.79	
**Sex**					0.185
Male	444	70.59	461	67.20	
Female	185	29.41	225	32.80	
**Tobacco use**					**<0.001**
Never	355	56.44	499	72.74	
Ever	274	43.56	187	27.26	
**Alcohol use**					**<0.001**
Never	428	68.04	526	76.68	
Ever	201	31.96	160	23.32	

^a^ Two-sided *χ*^2^ test and student t test; Bold values are statistically significant (*p* <0.05).

### Associations between *PADI4* tagging polymorphisms and risk of ESCC

**Figure 1:**
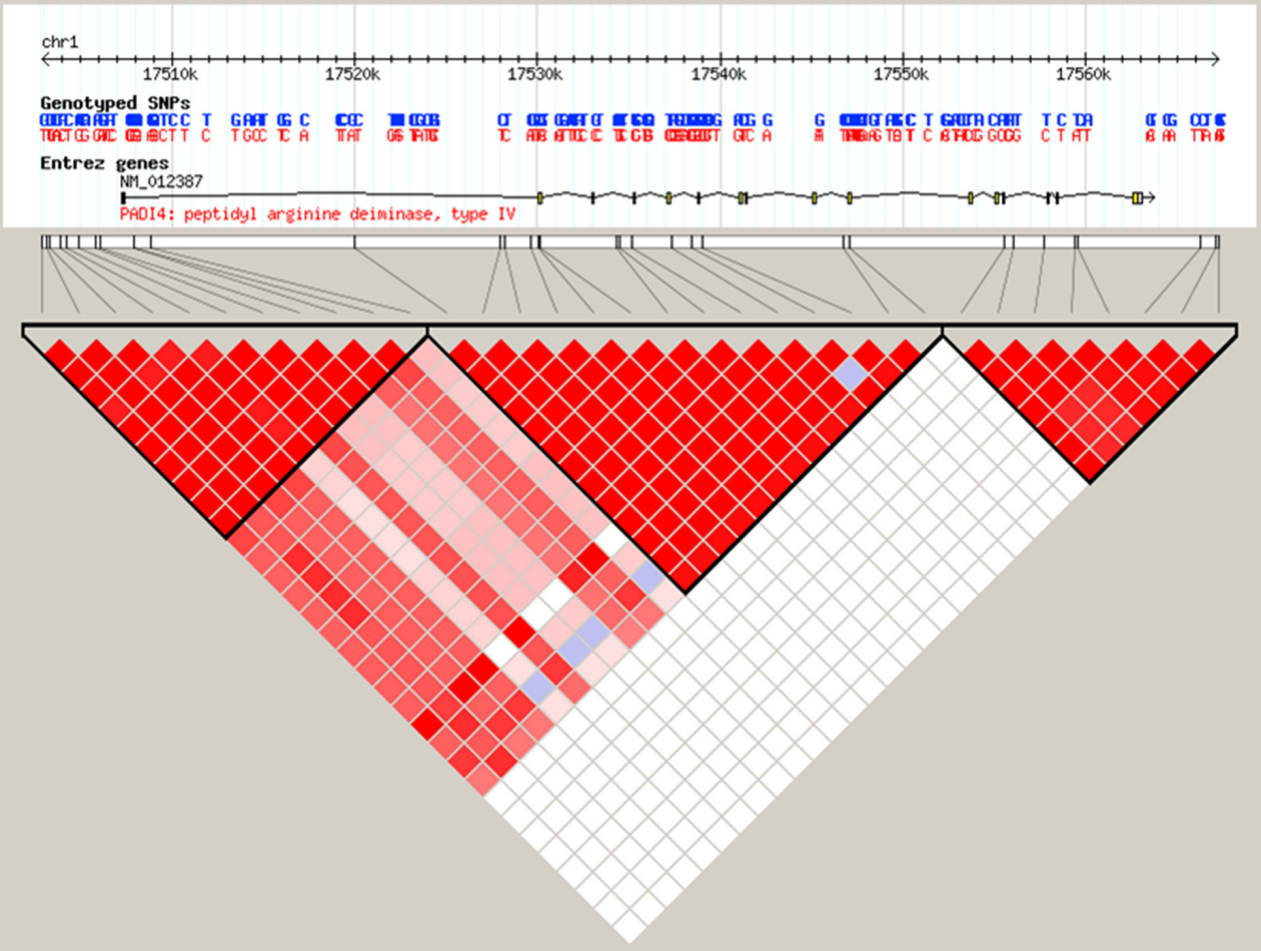
Linkage disequilibrium structure across the 50 kb region is represented, based on r2 coefficient calculated with the HapMap database The middle panel shows the genomic structure of the human *PADI4* gene. Exons are indicated by the vertical black bars. The genotyped tag SNPs are indicated with black bars. |D’| varies between 0 (no disequilibrium) and 1 (maximum disequilibrium), represented by shades of blue to white to pink to red. Blue:|D’| = 0 and red:|D’| = 1.

The seven tagging SNPs were selected on the basis of their pairwise linkage disequilibrium (LD) with the *r*^2^ threshold of 0.8 and minor allele frequency (MAF) ≥0.05 to capture all the common SNPs. Among eligible SNPs, linkage disequilibrium analysis was performed in the Chinese Han population (https://www.ncbi.nlm.nih.gov/variation/tools/1000genomes/), and the SNP loci with moderate correlation were chosen for further analyses. The LD structure across the *PADI4* genomic region was presented, and three blocks were defined (Figure 1). Next, we applied the “block-based” method, which exploits the principle of linkage disequilibrium observed within haplotype blocks, to search for tag SNPs. Several algorithms have been devised to partition chromosomal regions into haplotype blocks that are based on haplotype diversity, LD, four-gamete test and information complexity. We then used online database to predict the function of SNPs (http://www.regulomedb.org/) and selected seven tag SNPs for analysis (See Figure 1).

As shown in Table 2, the genotyping successful rates were ranging from 95.13% to 98.47%. In the control subjects, the genotype frequencies for these seven polymorphisms reached Hardy-Weinberg equilibrium (*p*-value for HWE, all *p*>0.05). The minor allele frequency (MAF) in our controls was comparable with the Chinese cohort in database for all seven SNPs loci.

**Table 2:** Primary information for *PADI4* rs11203366, rs1886302, rs1635562, rs1635564, rs16825533, rs2240337, rs2477137 polymorphisms

Genotyped SNPs	rs11203366	rs1886302	rs1635562	rs1635564	rs16825533	rs2240337	rs2477137
Ancestral Allele	G	T	A	C	A	G	C
Chromosome	1	1	1	1	1	1	1
Gene (ID)	PADI4 (23569)	PADI4 (23569)	PADI4 (23569)	PADI4 (23569)	PADI4 (23569)	PADI4 (23569)	PADI4 (23569)
Function	Missense	Intron region	Intron region	Intron region	Intron region	Intron region	Intergene region
Chr Pos (Genome Build 38.p7)	17331039	17308901	17360325	17357031	17339386	17347727	17304110
Regulome DB Score^a^	No Data	4	4	No Data	4	5	4
TFBS^b^	—	Y	—	—	—	—	Y
nsSNP	Y	—	—	—	—	—	—
MAF^c^ for Chinese in database	0.256	0.268	0.354	0.232	0.061	0.073	0.146
MAF in our controls (n = 608)	0.241	0.332	0.323	0.199	0.091	0.061	0.189
*p* value for HWE^d^ test in our controls	0.194	0.924	0.821	0.455	0.513	0.055	0.488
Genotyping method^e^	LDR	LDR	LDR	LDR	LDR	LDR	LDR
% Genotyping value	96.42%	96.80%	96.34%	95.13%	98.47%	95.13%	98.47%

^a^http://www.regulomedb.org/;

^b^TFBS: Transcription Factor Binding Site (https://snpinfo.niehs.nih.gov/cgi-bin/snpinfo/snpfunc.cgi);

^c^MAF: minor allele frequency;

^d^HWE: Hardy–Weinberg equilibrium;

^e^LDR: ligation detection reaction

The genotype distributions of *PADI4* SNPs in the cases and the controls are shown in Table 3. When the *PADI4* rs2240337 G>A SNP GG homozygote genotype (AA) was used as the reference group, both the GA heterozygote genotype (AB) and the AA mutated homozygote genotype (BB) were associated with a significantly decreased risk of ESCC (AB vs. AA: adjusted OR = 0.52, 95% CI = 0.39-0.71, *p*<0.0001; BB vs. AA: adjusted OR = 0.30, 95% CI = 0.13-0.68, *p* = 0.004). Logistic regression analyses revealed that the *PADI4* rs11203366 A>G, rs1886302 T>C, rs1635562 A>T, rs1635564 C>A, rs16825533 A>G, and rs2477137 C>A polymorphisms were not associated with the risk of ESCC. After the Bonferroni correction, for *PADI4* rs2240337 G>A, the *p*_adj_ = 0.031 for GA vs. GG after adjusted for age, sex, smoking and drinking status. *p*_adj_ < 0.001 for AA vs. GG. None of the rest 6 SNPs, showed significant associations with ESCC in this study population (*p*_adj_ > 0.05 in all comparison models).

**Table 3:** Main effects of *PADI4* SNPs on ESCC risk

Genotyped SNPs	Genotyping		AB vs. AA ^b^ Adjusted OR^c^ (95% CI); *p*	BB vs. AA Adjusted OR (95% CI); *p*	*p* trend
	Case (n=629) (AA/AB/BB) ^a^	Control (n=686) (AA/AB/BB)			
*PADI4*: rs11203366 A>G	219/293/103	214/301/138	1.00 (0.78–1.29);0.985	0.77 (0.56–1.07);0.117	0.128
*PADI4*: rs1886302 T>C	250/273/77	295/308/70	1.09 (0.86–1.39);0.487	1.37 (0.94–1.99);0.100	0.372
*PADI4*: rs1635562 A>T	295/251/64	302/285/70	0.90 (0.71–1.15);0.406	0.91 (0.62–1.34);0.632	0.682
*PADI4*: rs1635564 C>A	388/180/32	420/202/29	1.02 (0.80–1.31);0.860	1.22 (0.72–2.07);0.470	0.739
*PADI4*: rs16825533 A>G	528/85/6	560/109/7	0.86 (0.63–1.18);0.349	0.97 (0.32–2.98);0.957	0.477
*PADI4*: rs2240337 G>A	506/86/8	466/161/24	**0.52 (0.39–0.71);<0.0001**	**0.30 (0.13–0.68);0.004**	**<0.0001**
*PADI4*: rs2477137 C>A	399/202/18	447/202/27	1.15 (0.90–1.47);0.256	0.76 (0.40–1.41);0.381	0.365

^a^AA/AB/BB means homozygote, heterozygote and mutated homozygote; ^b^ Bonferroni correction was performed to correct the *p* value (*p*_*adj*_); For *PADI4*: rs2240337 G>A, the *p*_*adj*_ = 0.031 for GA vs. GG, *p*_*adj*_ < 0.001 for AA vs. GG, *p*_*adj*_ < 0.0001 for *p* trend. For the rest 6 SNPs, *p*_*adj*_> 0.05 in all comparison models; Bold values are statistically significant (*p* <0.05); ^c^ Adjusted for age, sex, smoking and drinking status.

### Associations between *PADI4* rs2240337 polymorphism and pathologic character of ESCC

Furthermore, we analyzed the correlation between *PADI4* rs2240337 G>A SNP and the clinic pathologic state. However, *PADI4* rs2240337 G>A SNP did not correlate with clinical tumor stage (*p* = 0.215) or grade (*p* = 0.497) (Table 4).

**Table 4:** Distribution of clinic pathologic characters by *PADI4* rs2240337 genotyping

	Genotyping			*Χ*^2^	*P*
	AA	AG	GG		
**Pathologic grade**					
1	4 (2.21%)	22 (12.15%)	155 (85.64%)	3.38	0.496
2	4 (1.18%)	53 (15.68%)	281 (83.14%)		
3	0 (0.00%)	11 (13.58%)	70 (86.42%)		
**Clinic stage**					
1	3 (2.52%)	14 (11.76%)	102 (85.71%)	8.34	0.215
2	1 (0.35%)	42 (14.63%)	244 (85.02%)		
3	2 (1.32%)	25 (16.56%)	124 (82.12%)		
4	2 (4.65%)	5 (11.63%)	36 (83.72%)		

### Stratification analyses of seven polymorphisms and risk of ESCC

To further evaluate the effects of these seven SNPs on the risk of ESCC according to different age, gender, smoking and alcohol drinking status, stratification analyses were performed as shown in Table 5–11. We showed that smoking significantly increased ESCC risk in *PADI4* rs11203366 AG/AA, rs1886302 CC/CT, rs1635562 AT, rs1635564 CA, rs2240337 AG and rs2477137 AC genotype. Alcohol drinking increased ESCC risk in *PADI4* rs11203366 AG, rs1635562 AT, rs1635564 CA, rs2477137 AC, rs1886302 CT genotype. In younger cohort (<63 years), *PADI4* rs16825533 AG genotype was associated with decreased risk of ESCC, while rs11203366 AA genotype was associated with increased risk of ESCC. In the non-drinking cohort, *PADI4* rs11203366 AA variant was associated with increased risk of ESCC. *PADI4* rs1886302 CC variant was associated with ESCC susceptibility in female cohort. In the non-alcohol drinking cohort, *PADI4* rs1886302 CC and CT variants were associated with decreased risk of ESCC. In rs1635562 TT subgroup, elder people (≥63 years) were more susceptible to ESCC.

**Table 5:** Stratified analyses between *PADI4* rs11203366 A>G polymorphism and ESCC risk by sex, age, smoking status and alcohol consumption

Variable	rs11203366 A>G (case/control) ^a^				Adjusted OR ^b^ (95%CI); *p*; *p*_h_^c^				
	GG	AG	AA	AG+AA	GG	AG	AA	AG+AA	AA vs. (GG+AG)
Sex									
Male	68/83	209/203	157/152	366/355	1.00	1.26(0.86-1.83); *p*:0.254 ; *p*_h_:0.304	1.26(0.85-1.86); *p*: 0.275; *p*_h_:0.879	1.26 (0.89-1.79); *p*: 0.211; *p*_h_:0.706	1.06 (0.81-1.41); *p*:0.67; *p*_h_:0.102
Female	35/55	84/98	62/62	146/160	1.00	1.35 (0.81-2.25); *p*:0.299 ; *p*_h_:0.304	1.57 (0.91-2.73); *p*:0.126 ; *p*_h_:0.879	1.43(0.89-2.32); *p*: 0.150; *p*_h_:0.706	0.78 (0.51-1.19); *p*:0.277 ; *p*_h_:0.102
Age									
<63	51/82	136/147	114/112	250/259	1.00	1.49(0.98-2.26); *p*:0.073 ; *p*_h_:0.555	1.64(1.06-2.53); ***p*:0.029** ; *p*_h_:0.953	1.56(1.05-2.29); ***p*: 0.032**; *p*_h_:0.676	0.80 (0.58-1.11); *p*:0.19; *p*_h_:0.102
≥63	52/56	157/154	105/102	262/256	1.00	1.09(0.71-1.70); *p*:0.740 ; *p*_h_:0.555	1.11(0.69-1.76); *p*:0.720 ; *p*_h_:0.953	1.10(0.73-1.67); *p*: 0.670; *p*_h_:0.676	0.97(0.69-1.35); *p*:0.865 ; *p*_h_:0.102
Smoking status									
Never	60/110	173/220	111/145	284/365	1.00	0.69(0.48-1.01); *p*:0.062 ; ***p***_**h**_**:0.000**	0.71(0.48-1.06); *p*:0.713 ; ***p***_**h**_**:0.000**	0.70(0.49-0.99); *p*: 0.055; *p*_h_:0.978	1.08(0.80-1.46); *p*:0.59 ; *p*_h_:0.124
Ever	43/28	120/81	108/69	228/150	1.00	1.04(0.59-1.8); *p*:1.000 ; ***p***_**h**_**:0.000**	0.98(0.56-1.72); *p*:1.000; ***p***_**h**_**:0.000**	1.01(0.60-1.69); *p*:1.000; *p*_h_:0.978	1.05(0.71-1.54); *p*:0.844; *p*_h_:0.124
Alcohol consumption									
Never	73/119	198/231	144/151	342/382	1.00	0.66(0.46-0.94); *p*:0.066**; *p***_**h**_**:0.013**	0.64(0.44-0.93); ***p*:0.020**; *p*_h_:0.283	0.69(0.50-0.95); ***p*:0.023**; *p*_h_:0.778	0.50(0.37-0.68); *p*:0.155; *p*_h_:0.146
Ever	30/19	95/70	75/63	170/133	1.00	1.16(0.61-2.23); *p*:0.742**; *p***_**h**_**:0.013**	1.33(0.68-2.58); *p*:0.503; *p*_h_:0.283	1.24(0.67-2.29); *p*:0.537; *p*_h_:0.778	0.85(0.55-1.30); *p*:0.509; *p*_h_:0.146

^a^ The genotyping success rate was 96.42% for rs11203366 A>G; ^b^ Adjusted for age, sex, smoking status and alcohol consumption (besides stratified factors accordingly) in a logistic regression model; ^c^ *p*_h_ for heterogeneity; Bold values are statistically significant (*p*<0.05).

*PADI4* rs11203366 variant AA was associated with ESCC among younger patients (<63 years) (*p*=0.029). In the dominant model, *PADI4* rs11203366 was associated with ESCC among younger patients (<63 years) (*p*=0.032). In the cohort of subjects who carry *PADI4* rs11203366 AG variant or AA variant, smoking significantly increased the ESCC susceptibility (*p*_h_=0.000).

In the non-alcohol drinking cohort, *PADI4* rs11203366 AA (*p*=0.020) variant was associated with increased risk of ESCC.

In the dominant (*p*=0.023) model, *PADI4* rs11203366 A>G was associated with increased risk of ESCC.

In the *PADI4* rs11203366 AG subgroup, alcohol drinking significantly increased the risk of ESCC (*p*_h_=0.013).

**Table 6:** Stratified analyses between *PADI4* rs1886302 T>C polymorphism and ESCC risk by sex, age, smoking status and alcohol consumption

Variable	rs1886302 T>C (case/control) ^a^				Adjusted OR ^b^ (95%CI); *p*; *p*_h_^c^				
	TT	CT	CC	CT+CC	TT	CT	CC	CT+CC	CC vs. (TT+CT)
Sex									
Male	187/198	197/207	44/44	241/251	1.0	0.99(0.75-1.31); *p*:1.000; *p*_h_:0.196	0.94(0.59-1.50); *p*:0.814; *p*_h_:0.481	0.98(0.75-1.28); *p*:0.946; *p*_h_: 0.419	1.05(0.68-1.64); *p*:0.823; ***p***_**h**_**: 0.022**
Female	63/97	76/101	33/26	109/127	1.0	0.86(0.56-1.33); *p*:0.580; *p*_h_: 0.196	0.51(0.28-0.94); ***p*:0.032**; *p*_h_:0.481	0.76(0.50-1.14); *p*:0.215; *p*_h_: 0.419	1.81(1.04-3.16); ***p*:0.046; *p***_h_**: 0.022**
Age									
<63	142/166	121/153	31/37	152/190	1.0	1.08(0.78-1.50); *p*:0.677; *p*_h_:0.197	1.02(0.60-1.73); *p*:1.000; *p*_h_:0.127	1.07(0.79-1.46); *p*:0.694; *p*_h_:0.066	1.02(0.61-1.68); *p*:1.000; *p*_h_:0.398
≥63	108/129	152/155	46/33	198/188	1.0	0.85(0.61-1.19); *p*:0.39; *p*_h_:0.197	0.60(0.36-1.00); *p*:0.068; *p*_h_:0.127	0.79(0.58-1.09); *p*:0.187; *p*_h_: 0.066	1.52(0.94-2.46); *p*:0.092; *p*_h_:0.398
Smoking status									
Never	125/207	159/229	49/56	208/285	1.0	0.87(0.64-1.17); *p*:0.400; ***p***_**h**_**:0.000**	0.69(0.44-1.07); *p*:0.110; ***p***_**h**_**:0.030**	0.83(0.62-1.10); *p*:0.219; ***p***_**h**_**:0.000**	0.74(0.49-1.12); *p*:0.167; ***p***_**h**_**:0.000**
Ever	125/88	114/79	28/14	142/93	1.0	0.98(0.66-1.46); *p*:1.000; ***p***_**h**_**:0.000**	0.71(0.35-1.43); *p*:0.39; ***p***_**h**_**:0.030**	0.93(0.64-1.36); *p*:0.77; ***p***_**h**_**:0.000**	1.39(0.71-2.74); *p*:0.409; ***p***_**h**_**:0.000**
Alcohol consumption									
Never	157/61	188/238	59/61	247/299	1.0	3.26(2.29-4.63); ***p*:0.000**; ***p***_**h**_**:0.023**	2.66(1.67-4.23); ***p*:0.000**; *p*_h_:0.104	3.12(2.22-4.38); ***p*:0.000; *p***_h_**:0.006**	0.84(0.57-1.24); *p*:0.426; ***p***_**h**_**: 0.000**
Ever	93/75	85/70	18/9	103/79	1.0	1.02(0.66-1.58); *p*:1.000; ***p***_**h**_**:0.023**	0.62(0.26-1.46); *p*:0.302; *p*_h_:0.104	0.95(0.62-1.45); *p*:0.83; ***p***_**h**_**:0.006**	1.63(0.71-3.74); *p*:0.314; ***p***_**h**_**: 0.000**

^a^ The genotyping success rate was 96.80% for rs1886302 T>C; ^b^ Adjusted for age, sex, smoking status and alcohol consumption (besides stratified factors accordingly) in a logistic regression model; ^c^ *p*_h_ for heterogeneity; Bold values are statistically significant (*p*<0.05).

*PADI4* rs1886302 variant CC was associated with ESCC susceptibility in female cohort (*p*=0.032). In the recessive model, *PADI4* rs1886302 was associated with ESCC susceptibility in females (*p*=0.046). In the recessive model, male cohort has a significantly higher risk than females (*p*_h_=0.022).

Smoking significantly increased ESCC susceptibility in both CC (*p*_h_=0.000) and CT (*p*_h_=0.030) genotypes. Smoking is associated with increased risk of ESCC in both dominant and recessive models.

In the non-alcohol drinking cohort, *PADI4* rs1886302 variant CC and CT variants were associated with decreased risk of ESCC (*p*=0.000, respectively), in the dominant model, *PADI4* rs1886302 T>C was associated with decreased risk of ESCC (*p*=0.000).

Among *PADI4* rs1886302 CT subgroup, alcohol drinking significantly increased the risk of ESCC (*p*_h_=0.023). In both the *PADI4* rs1886302 T>C polymorphism dominant (*p*_h_=0.006) and recessive (*p*_h_=0.000) models, alcohol drinking significantly increased ESCC susceptibility.

**Table 7:** Stratified analyses between *PADI4* rs1635562 A>T polymorphism and ESCC risk by sex, age, smoking status and alcohol consumption

Variable	rs1635562 A>T (case/control) ^a^				Adjusted OR ^b^ (95%CI); *p*; *p*_h_^c^				
	AA	AT	TT	AT+TT	AA	AT	TT	AT+TT	TT vs. (AA+AT)
Sex									
Male	206/196	178/201	44/47	222/248	1.00	1.18(0.89-1.57); *p*:0.252; *p*_h_:0.921	1.12(0.71-1.77); *p*:0.644; *p*_h_:0.842	1.19(0.89-1.57); *p*:0.252; *p*_h_:0.862	0.97(0.63-1.49); *p*:0.912; *p*_h_:0.327
Female	89/106	73/84	20/23	93/107	1.00	0.96(0.63-1.47); *p*:0.914; *p*_h_:0.921	0.96(0.49-1.87); *p*:1.000; *p*_h_:0.842	0.96(0.65-1.44); *p*:0.920; *p*_h_:0.862	0.98(0.52-1.85); *p*:0.981; *p*_h_: 0.327
Age									
<63	148/157	134/155	19/35	153/190	1.00	1.09(0.79-1.51); *p*:0.622; *p*_h_:0.817	1.74(0.95-3.17); *p*:0.077; ***p***_**h**_**:0.018**	1.17(0.86-1.59); *p*:0.344; *p*_h_:0.201	0.49(0.27-0.88); *p*:0.089; *p*_h_:0.613
≥63	147/145	117/130	45/35	162/165	1.00	1.13(0.80-1.58); *p*:0.55; *p*_h_:0.817	0.79(0.48-1.29); *p*:0.378; ***p***_**h**_**: 0.018**	1.03(0.75-0.95); *p*:0.872; *p*_h_:0.201	1.34(0.84-2.15); *p*:0.233; *p*_h_: 0.613
Smoking status									
Never	167/219	141/206	38/49	179/255	1.00	1.11(0.83-1.49); *p*:0.500; ***p***_**h**_**:0.000**	0.98(0.62-1.57); *p*:1.000; *p*_h_:0.199	1.09(0.82-1.43); *p*:0.572; ***p***_**h**_**:0.000**	0.99(0.62-1.56); *p*:1.000; ***p***_**h**_**: 0.000**
Ever	128/83	110/79	26/31	136/110	1.00	1.11(0.74-1.65); *p*:0.683; ***p***_**h**_**:0.000**	1.84(1.02-3.32); *p*:0.05; *p*_h_:0.199	1.25(0.86-1.81); *p*:0.256; ***p***_**h**_**:0.000**	0.57(0.33-0.99); *p*:0.062; ***p***_**h**_**: 0.000**
Alcohol consumption									
Never	194/230	172/218	49/52	221/270	1.00	1.07(0.81-1.41); *p*:0.672; ***p***_**h**_**:0.039**	0.89(0.58-1.38); *p*:0.658; *p*_h_:0.760	0.03(0.79-1.34); *p*:0.842; *p*_h_:0.086	1.15(0.76-1.75); *p*:0.526; ***p***_**h**_**:0.001**
Ever	101/72	79/67	15/18	91/85	1.00	1.19(0.76-1.86); *p*:0.497; ***p***_**h**_**:0.039**	1.68(0.79-3.56); *p*:0.185; *p*_h_:0.760	1.31(0.86-2.00); *p*:0.237; *p*_h_: 0.086	0.64(0.31-1.32); *p*:0.271; ***p***_**h**_**:0.001**

^a^ The genotyping success rate was 96.34% for rs1635562 A>T; ^b^ Adjusted for age, sex, smoking status and alcohol consumption (besides stratified factors accordingly) in a logistic regression model; ^c^ *p*_h_ for heterogeneity; Bold values are statistically significant (*p*<0.05).

In *PADI4* rs1635562 TT genotype, elder people (≥63 years) were more susceptible to ESCC (*p*_h_=0.018).

Smoking significantly increased ESCC susceptibility in AT (*p*_h_=0.000) genotype. Smoking is associated with increased risk of ESCC in both dominant and recessive models.

Alcohol drinking significantly increased ESCC susceptibility in AT (*p*_h_=0.039) genotype. Alcohol drinking is associated with increased risk of ESCC in recessive model.

**Table 8:** Stratified analyses between *PADI4* rs1635564 C>A polymorphism and ESCC risk by sex, age, smoking status and alcohol consumption

Variable	rs1635564 C>A (case/control) ^a^				Adjusted OR ^b^ (95%CI); *p*; *p*_h_^c^				
	CC	CA	AA	CA+AA	CC	CA	AA	CA+AA	AA vs. (CC+CA)
Sex									
Male	269/283	132/134	25/18	157/152	1.00	0.96(0.72-1.29); *p*:0.823; *p*_h_:0.138	0.68(0.37-1.28); *p*:0.269; *p*_h_:0.174	0.92(0.69-1.22); *p*:0.570; *p*_h_:0.059	1.44(0.78-2.69); *p*:0.275; *p*_h_:0.186
Female	119/137	48/68	7/11	55/79	1.00	1.23(0.79-1.92); *p*:0.370; *p*_h_:0.138	1.37(0.51-3.16); *p*:0.628; *p*_h_:0.174	1.25(0.82-1.90); *p*:0.335; *p*_h_:0.059	0.78(0.29-2.06); *p*:0.809; *p*_h_:0.186
Age									
<63	183/215	93/116	18/14	111/130	1.00	1.06(0.76-1.49); *p*:0.732; *p*_h_:0.259	0.66(0.32-1.37); *p*:0.275; *p*_h_:0.543	0.99(0.27-1.37); *p*:1.000; *p*_h_:0.408	1.54(0.75-3.16); *p*:0.278; *p*_h_:0.111
≥63	205/205	87/86	14/15	101/101	1.00	0.99(0.69-1.41); *p*:1.000; *p*_h_:0.259	1.07(0.50-2.28); *p*: 1.000; *p*_h_:0.543	1.00(0.71-1.40); *p*: 1.000; *p*_h_:0.408	0.93(0.44-1.96); *p*:0.852; *p*_h_:0.111
Smoking status									
Never	212/297	106/155	19/20	125/175	1.00	1.04(0.77-1.41); *p*:0.817; ***p***_**h**_**:0.000**	0.75(0.39-1.44); *p*:0.404; *p*_h_:0.437	0.99(0.75-1.34); *p*:1.000; ***p***_**h**_**:0.000**	1.35(0.71-2.57); *p*:0.406; ***p***_**h**_**:0.000**
Ever	176/123	74/47	13/9	87/56	1.00	0.91(0.59-1.40); *p*:0.742; ***p***_**h**_**:0.000**	0.99(0.41-2.39); *p*:1.000; *p*_h_:0.437	0.92(0.61-1.38); *p*:0.756; ***p***_**h**_**:0.000**	0.98(0.41-2.35); *p*:1.000; ***p***_**h**_**:0.000**
Alcohol consumption									
Never	265/314	121/161	21/24	142/185	1.00	1.12(0.84-1.49); *p*:0.465; ***p***_**h**_**:0.006**	0.96(0.53-1.77); *p*:1.000; *p*_h_:0.135	1.10(0.84-1.45); *p*:0.532; ***p***_**h**_**:0.002**	0.93(0.51-1.69); *p*:0.878; ***p***_**h**_**:0.001**
Ever	123/106	59/41	11/5	70/46	1.00	0.81(0.50-1.29); *p*:0.400; ***p***_**h**_**:0.006**	0.53(0.18-1.57); *p*:0.304; *p*_h_:0.135	0.76(0.48-1.20); *p*:0.253; ***p***_**h**_**:0.002**	1.78(0.60-5.23); *p*:0.317; ***p***_**h**_**:0.001**

^a^ The genotyping success rate was 95.13% for rs1635564 C>A; ^b^ Adjusted for age, sex, smoking status and alcohol consumption (besides stratified factors accordingly) in a logistic regression model; ^c^ *p*_h_ for heterogeneity; Bold values are statistically significant (*p*<0.05).

*PADI4* rs1635564 polymorphism was not associated with the ESCC susceptibility. However, in rs1635564 CA genotype, smoking significantly increased risk of ESCC (*p*_h_=0.000). Smoking increased ESCC susceptibility in both dominant and recessive models (*p*_h_=0.000, respectively). In rs1635564 CA genotype, alcohol drinking significantly increased risk of ESCC (*p*_h_=0.006). Alcohol drinking increased ESCC susceptibility in both dominant and recessive models (*p*_h_=0.002,0.001, respectively).

**Table 9:** Stratified analyses between *PADI4* rs16825533 A>G polymorphism and ESCC risk by sex, age, smoking status and alcohol consumption

Variable	rs16825533 A>G (case/control) ^a^				Adjusted OR ^b^ (95%CI); *p*; *p*_h_^c^				
	AA	AG	GG	AG+GG	AA	AG	GG	AG+GG	GG vs. (AA+AG)
Sex									
Male	379/378	53/73	5/2	58/75	1.00	1.38(0.94-2.02); *p*:0.102; *p*_h_:0.504	0.40(0.07-2.08); *p*:0.451; *p*_h_:0.067	1.29(0.89-1.88); *p*:0.188; *p*_h_:0.891	2.61(0.50-13.52); *p*:0.279; *p*_h_:0.237
Female	149/182	32/36	1/5	33/41	1.00	0.921 (0.55-1.56); *p*: 0.790; *p*_h_:0.504	0.41(0.47-35.42); *p*:0.232; *p*_h_:0.067	1.02(0.61-1.69); *p*:1.000; *p*_h_:0.891	0.24(0.28-2.08); *p*:0.230; *p*_h_:0.237
Age									
<63	261/286	36/68	4/3	40/71	1.00	1.72(1.11-2.67); ***p*:0.018**; ***p***_**h**_**:0.006**	0.68(0.15-3.08); *p*:0.715; *p*_h_:0.396	1.62(1.06-2.47); ***p*:.0.028**; ***p***_**h**_**:0.014**	1.59(0.35-7.16); *p*:0.708; *p*_h_:0.110
≥63	267/274	49/41	2/4	51/45	1.00	0.82(0.52-1.28); *p*:0.426; ***p***_**h**_**:0.006**	1.95(0.35-10.73); *p*:0.686; *p*_h_:0.396	0.860(0.56-1.33); *p*:0.508; ***p***_**h**_**:0.014**	0.49(0.09-2.74); *p*:0.686; *p*_h_:0.110
Smoking status									
Never	290/402	53/82	4/6	57/88	1.00	1.12(0.77-1.63); *p*:0.633; *p*_h_:0.054	1.08(0.30-3.87); *p*:1.000; *p*_h_:0.427	1.11(0.77-1.61); *p*:0.579; ***p***_**h**_**:0.040**	0.94(0.26-3.36); *p*:1.000; ***p***_**h**_**:0.000**
Ever	238/158	32/27	2/1	34/28	1.00	1.27 (0.73-2.20); *p*:0.398; *p*_h_:0.054	0.75(0.68-8.38); *p*:1.000; *p*_h_:0.427	1.24 (0.72-2.13); *p*:0.487; ***p***_**h**_**:0.040**	1.37(0.12-15.22); *p*:1.000; ***p***_**h**_**:0.000**
Alcohol consumption									
Never	355/423	59/87	4/7	63/94	1.00	1.24(0.86-1.77); *p*:0.277; *p*_h_:0.098	1.47(0.43-5.06); *p*:0.762; *p*_h_:0.999	1.25(0.88-1.78); *p*:0.219; *p*_h_:0.051	0.70(0.21-2.42); *p*:0.763; ***p***_**h**_**:0.001**
Ever	173/137	26/22	2/0	28/22	1.00	1.07(0.58-1.97); *p*:0.877; *p*_h_:0.098	1.01(0.99-1.03); *p*:0.506; *p*_h_:0.999	0.99(0.54-1.81); *p*:1.000; *p*_h_:0.051	1.01(0.99-1.02); *p*:0.505; ***p***_**h**_**:0.001**

^a^ The genotyping success rate was 98.47% for rs16825533 A>G; ^b^ Adjusted for age, sex, smoking status and alcohol consumption (besides stratified factors accordingly) in a logistic regression model; ^c^ *p*_h_ for heterogeneity; Bold values are statistically significant (*p*<0.05).

*PADI4* rs16825533 A>G polymorphism was not associated with the ESCC susceptibility. However, in younger cohort (<63 years), rs16825533 AG genotype was associated with decreased risk of ESCC (*p*=0.018). In younger cohort (<63 years), *PADI4* rs16825533 A>G polymorphism was associated with decreased risk of ESCC in the dominant model (*p*=0.028). In both the *PADI4* rs16825533 AG genotype (*p*_h_=0.006) and the dominant model (*p*_h_=0.014), younger cohort (<63 years) had lower susceptibility to ESCC.

Smoking increased ESCC susceptibility in both dominant (*p*_h_=0.040) and recessive (*p*_h_=0.000) models. Alcohol drinking increased ESCC susceptibility in the recessive model (*p*_h_=0.001).

**Table 10:** Stratified analyses between *PADI4* polymorphism rs2240337 G>A and ESCC risk by sex, age, smoking status and alcohol consumption

Variable	rs2240337 G>A (case/control) ^a^				Adjusted OR ^b^ (95%CI); *p*; *p*_h_^c^				
	GG	AG	AA	AG+AA	GG	AG	AA	AG+AA	AA vs. (GG+AG)
Sex									
Male	366/319	54/99	6/17	60/116	1.00	2.10(1.46-3.03); ***p*:0.000**; *p*_h_:0.841	3.25(1.27-8.35); ***p*:0.011**; *p*_h_:0.821	2.22(1.57-3.14); ***p*:0.000;** *p*_h_:0.854	0.35(0.14-0.90); ***p*:0.033**; *p*_h_:0.107
Female	140/147	32/62	2/7	34/69	1.00	1.85(1.14-2.99); ***p*:0.017**; *p*_h_:0.841	3.33(0.68-16.32); *p*:0.176; *p*_h_:0.821	0.47(0.29-0.75); ***p*:0.002;** *p*_h_:0.854	0.35(0.07-1.69); *p*:0.309; *p*_h_:0.107
Age									
<63	261/253	29/84	4/8	33/92	1.00	1.71(1.04-2.79); ***p*:0.037; *p***_h_**: 0.006**	2.06(0.61-6.54); *p*:0.258; *p*_h_:0.403	2.88(1.86-4.44); ***p*:0.000; *p***_h_**:0.021**	0.58(0.17-0.95); *p*:0.561; *p*_h_:0.097
≥63	245/213	57/77	4/16	61/93	1.00	1.55(1.05-2.29); ***p*:0.031; *p***_h_**: 0.006**	4.60(1.52-13.97); ***p*:0.005**; *p*_h_:0.403	1.75(1.21-2.54); ***p*:0.004; *p***_h_**:0.021**	0.24(0.08-0.73); ***p*:0.011**; *p*_h_:0.097
Smoking status									
Never	275/324	58/130	4/18	62/148	1.00	1.9(1.34-2.69); ***p*:0.000; *p***_h_**:0.021**	3.82(1.28-11.42); ***p*:0.015**; *p*_h_:0.196	2.03(1.45-2.84); ***p*:0.000; *p***_h_**:0.011**	0.30(0.10-0.90); ***p*:0.027; *p***_h_**:0.000**
Ever	231/142	28/31	4/6	32/37	1.00	1.80(1.04-3.13); ***p*:0.045; *p***_h_**:0.021**	2.44(0.68-8.79); *p*:0.194; *p*_h_:0.196	1.88(1.12-3.16); ***p*:0.023; *p***_h_**:0.011**	0.45(0.12-0.60); *p*:0.328; ***p***_**h**_**:0.000**
Alcohol consumption									
Never	336/346	64/134	7/19	71/153	1.00	2.03(1.46-2.84); ***p*:0.000**; *p*_h_:0.100	2.64(1.09-6.35); ***p*:0.028**; *p*_h_:0.605	2.09(1.52-2.88); ***p*:0.000**; *p*_h_:0.157	0.44(0.18-1.06); *p*:0.072; ***ph:* 0.000**
Ever	170/120	22/27	1/5	23/32	1.00	1.74(0.95-3.20); ***p*:0.087**; *p*_h_:0.100	7.08(0.82-61.4); ***p*:0.086**; *p*_h_:0.605	1.97(1.09-3.54); ***p*:0.026**; *p*_h_:0.157	0.15(0.02-1.33); *p*:0.091; ***p***_**h**_**: 0.000**

^a^ The genotyping success rate was 95.13% for rs2240337 G>A; ^b^ Adjusted for age, sex, smoking status and alcohol consumption (besides stratified factors accordingly) in a logistic regression model; ^c^ *p*_h_ for heterogeneity; Bold values are statistically significant (*p*<0.05).

*PADI4* rs2240337 G>A polymorphism was associated with the ESCC susceptibility. In rs2240337 AG genotype, elder cohort (≥ 63 years) had increased susceptibility to ESCC (*p*_h_=0.006). Elder age was associated with ESCC risk in the dominant model (*p*_h_=0.021). In rs2240337 AG genotype, smoking increased susceptibility to ESCC (*p*_h_=0.021). Smoking was associated with ESCC risk in the dominant (*p*_h_=0.011) and recessive (*p*_h_=0.000) models. Alcohol drinking increased ESCC susceptibility in the recessive model (*p*_h_=0.000).

**Table 11:** Stratified analyses between *PADI4* polymorphism rs2477137 and ESCC risk by sex, age, smoking status and alcohol consumption

Variable	rs2477137 C>A (case/control) ^a^				Adjusted OR ^b^ (95%CI); *p*; *p*_h_^c^				
	CC	AC	AA	AC+AA	CC	AC	AA	AC+AA	AA vs. (CC+AC)
Sex									
Male	290/300	137/137	10/16	147/153	1.00	0.97(0.73-1.28); *p*:0.827; *p*_h_:1.000	1.55(0.69-3.46); *p*:0.321; *p*_h_:0.805	1.00(0.76-1.33); *p*:1.000; *p*_h_:0.999	1.56(0.70-3.48); *p*:0.322; *p*_h_:0.156
Female	109/147	65/65	8/11	73/76	1.00	0.74(0.48-1.13); *p*:0.194; *p*_h_: 1.000	1.02(0.39-2.62); *p*:1.000; *p*_h_:0.805	0.77(0.52-1.16); *p*:0.216; *p*_h_:0.999	1.13(0.44-2.87); *p*:1.000; *p*_h_:0.156
Age									
<63	210/241	86/104	5/12	91/116	1.00	1.05(0.75-1.48); *p*:0.795; *p*_h_:0.073	2.09(0.73-6.03); *p*:0.217; *p*_h_:0.262	1.11(0.79-1.55); *p*:0.556; ***p***_**h**_**:0.049**	0.49(0.17-1.39); *p*:0.220; *p*_h_:0.167
≥63	189/206	116/98	13/15	129/113	1.00	0.78(0.56-1.08); *p*:0.149; *p*_h_:0.073	1.06(0.49-2.28); *p*:1.000; *p*_h_:0.262	0.80(0.58-1.11); *p*:0.192; ***p***_**h**_**:0.049**	0.86(0.40-1.85); *p*:0.847; *p*_h_:0.167
Smoking status									
Never	216/317	119/151	12/22	131/173	1.00	0.87(0.64-1.16); *p*:0.363; ***p***_**h**_**:0.001**	1.25(0.61-2.58); *p*:0.593; *p*_h_:0.263	0.90(0.68-1.19); *p*:0.512; ***p***_**h**_**:0.000**	0.76(0.37-1.56); *p*:0.484; ***p***_**h**_**:0.000**
Ever	183/130	83/51	6/5	89/56	1.00	0.86(0.57-1.31); *p*:0.529; ***p***_**h**_**:0.001**	1.17(0.35-3.93); *p*:1.000; *p*_h_:0.263	0.89(0.59-1.33); *p*:0.61; ***p***_**h**_**:0.000**	0.82(0.25-2.72); *p*:0.746; ***p***_**h**_**:0.000**
Alcohol consumption									
Never	264/337	138/156	16/24	154/180	1.00	0.88(0.67-1.17); *p*:0.431; ***p***_**h**_**:0.045**	1.18(0.61-2.26); *p*:0.742; *p*_h_:1.000	0.92(0.70-1.19); *p*:0.537; ***p***_**h**_**:0.038**	0.82(0.43-1.56); *p*:0.627; ***p***_**h**_**:0.000**
Ever	135/110	64/46	2/3	66/49	1.00	0.88(0.56-1.39); *p*:0.64; ***p***_**h**_**:0.045**	1.84(0.30-11.21); *p*:0.661; *p*_h_:1.000	0.91(0.58-1.43); *p*:0.733; ***p***_**h**_**:0.038**	0.52(0.09-3.17); *p*:0.658; ***p***_**h**_**:0.000**

^a^ The genotyping success rate was 98.47% for rs2477137 C>A; ^b^ Adjusted for age, sex, smoking status and alcohol consumption (besides stratified factors accordingly) in a logistic regression model; ^c^ *p*_h_ for heterogeneity; Bold values are statistically significant (*p*<0.05).

*PADI4* rs2477137 C>A polymorphism was not associated with the ESCC susceptibility. Elder cohort (≥ 63 years) had increased susceptibility to ESCC in the dominant model (*p*_h_=0.049). In rs2477137 AC genotype, smoking increased susceptibility to ESCC (*p*_h_=0.001). Smoking was associated with ESCC risk in the dominant (*p*_h_=0.000) and recessive (*p*_h_=0.000) models. In rs2477137 AC genotype, alcohol drinking increased susceptibility to ESCC (*p*_h_=0.045). Alcohol drinking was associated with ESCC risk in the dominant (*p*_h_=0.038) and recessive (*p*_h_=0.000) models.

### Linkage disequilibrium analyses and association test

**Figure 2:**
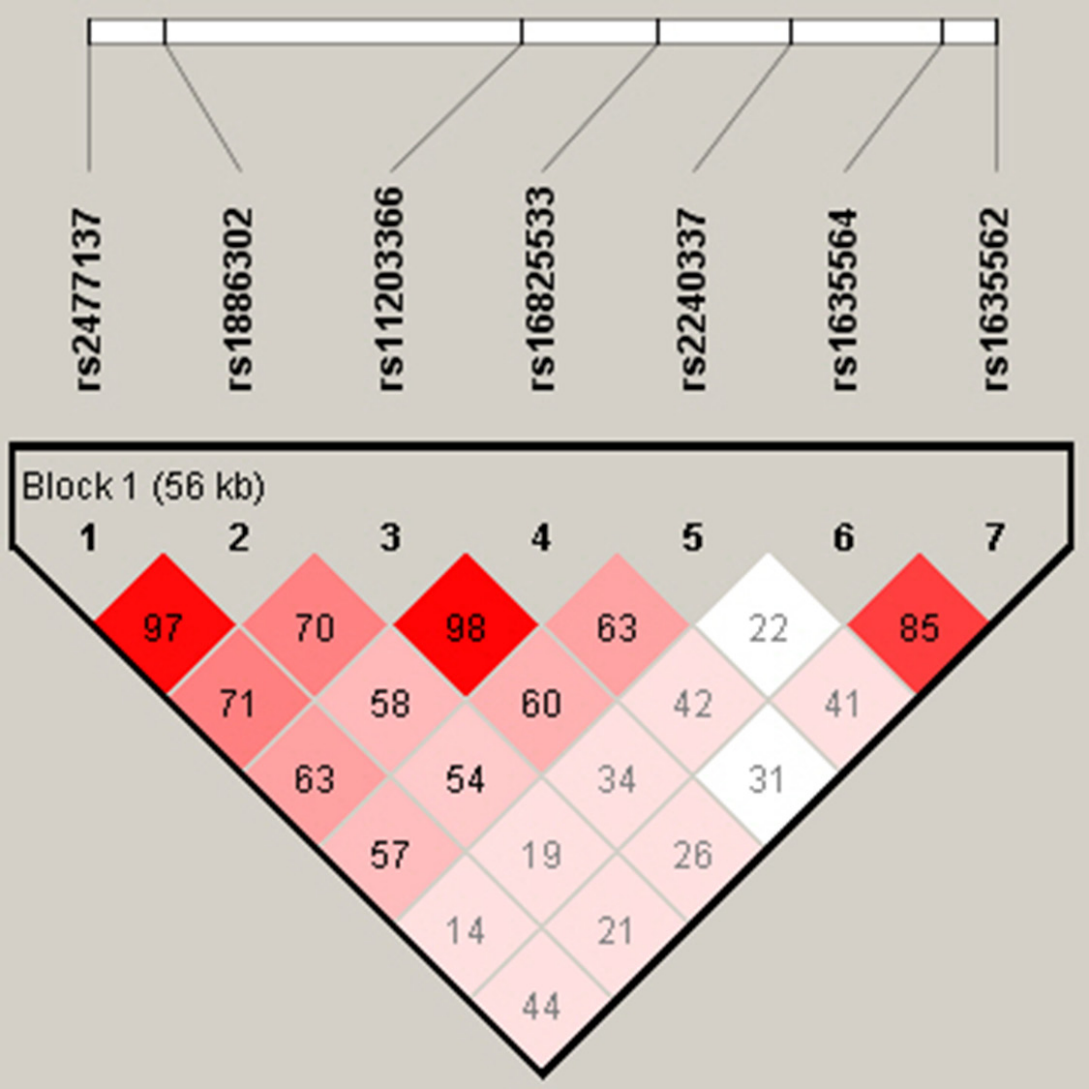
Association test of seven *PADI4* SNPs (by Haploview Software, V 4. 2) There are associations between these seven loci.

Linkage disequilibrium analyses in both controls and cases were conducted as shown in Table 12–13, there were correlations between these seven loci. Association test was performed using Haploview software (v 4.2), there were associations between these seven loci (Figure 2).

**Table 12:** Linkage disequilibrium analyses of *PADI4* rs11203366, rs1886302, rs1635562, rs1635564, rs16825533, rs2240337, rs2477137 in control group

*D*‘:	rs1886302	rs11203366	rs16825533	rs2240337	rs1635564	rs1635562
rs2477137	**1**	**0.803**	0.571	0.485	0.211	0.47
rs1886302	-	**0.764**	0.509	0.508	0.211	0.209
rs11203366	-	-	**0.978**	0.525	0.445	0.28
rs16825533	-	-	-	0.485	0.5	0.509
rs2240337	-	-	-	-	0.039	0.303
rs1635564	-	-	-	-	-	**0.836**

*r*^2^:	rs1886302	rs11203366	rs16825533	rs2240337	rs1635564	rs1635562
rs2477137	**0.464**	0.19	0.139	0.19	0.042	0.025
rs1886302	-	**0.367**	0.051	0.097	0.022	0.011
rs11203366	-	-	0.124	0.065	0.065	0.03
rs16825533	-	-	-	0.004	0.099	0.012
rs2240337	-	-	-	-	0	0.009
rs1635564	-	-	-	-	-	0.084

*D*'>0, *r*^2^>0: There were linkage disequilibrium correlations among different loci; *D*'>0.7, *r*^2^>0.3: there were closer linkage disequilibrium correlation among different loci.

**Table 13:** Linkage disequilibrium analyses of *PADI4* rs11203366, rs1886302, rs1635562, rs1635564, rs16825533, rs2240337, rs2477137 in case group

*D*‘:	rs1886302	rs11203366	rs16825533	rs2240337	rs1635564	rs1635562
rs2477137	**0.942**	0.619	**0.717**	**0.766**	0.085	0.42
rs1886302	-	0.658	0.687	0.653	0.18	0.214
rs11203366	-	-	**1**	**0.748**	0.245	0.251
rs16825533	-	-	-	**0.997**	0.319	0.07
rs2240337	-	-	-	-	0.445	0.661
rs1635564	-	-	-	-	-	**0.879**

*r*^2^:	rs1886302	rs11203366	rs16825533	rs2240337	rs1635564	rs1635562
rs2477137	**0.381**	0.133	0.184	0.232	0.007	0.019
rs1886302	-	**0.341**	0.072	0.072	0.015	0.011
rs11203366	-	-	0.123	0.078	0.023	0.019
rs16825533	-	-	-	0.008	0.034	0
rs2240337	-	-	-	-	0.005	0.018
rs1635564	-	-	-	-	-	0.089

*D*'>0, *r*^2^>0: There were linkage disequilibrium correlations among different loci; *D*'>0.7, *r*^2^>0.3: there were closer linkage disequilibrium correlation among different loci.

### Haplotype analyses of *PADI4* polymorphisms and susceptibility to ESCC

As shown in Table 14, haplotype analyses showed that *PADI4* C_rs2477137_T_rs1886302_A_rs11203366_A_rs1635564_G_rs2240337_C_rs1635564_A_rs1635562_ was the most common haplotype in both groups (24.5% in controls, 25.5% in cases). The haplotype *PADI4* **A**_rs2477137_**C**_rs1886302_**G**_rs11203366_**G**_rs16825533_**G**_rs2240337_**A**_rs1635564_**A**_rs1635562_ frequency and *PADI4* **C**_rs2477137_**T**_rs1886302_**G**_rs11203366_**A**_rs1635564_**G**_rs2240337_**C**_rs1635564_**T**_rs1635562_ frequency were significantly lower in ESCC cases as compared with controls (0.019 vs. 0.036, *p*=0.007; 0.019 vs. 0.031, *p*=0.038, respectively), suggesting that both *PADI4* **A**_rs2477137_**C**_rs1886302_**G**_rs11203366_**G**_rs16825533_**G**_rs2240337_**A**_rs1635564_**A**_rs1635562_ and *PADI4* **C**_rs2477137_**T**_rs1886302_**G**_rs11203366_**A**_rs1635564_**G**_rs2240337_**C**_rs1635564_**T**_rs1635562_ haplotypes may be correlated with decreased susceptibility of ESCC (OR=0.491, 95%CI:0.290-0.831; OR=0.568, 95%CI:0.330-0.975, respectively). Haplotype *PADI4* **C**_rs2477137_**T**_rs1886302_**A**_rs11203366_**A**_rs1635564_**G**_rs2240337_**A**_rs1635564_**A**_rs1635562_ frequency was significantly higher in ESCC cases as compared with controls (0.073 vs. 0.049, *p*=0.042), suggesting that haplotype *PADI4* **C**_rs2477137_**T**_rs1886302_**A**_rs11203366_**A**_rs1635564_**G**_rs2240337_**A**_rs1635564_**A**_rs1635562_ genetic polymorphism may be correlated with increased susceptibility of ESCC (OR=1.435, 95%CI: 1.011-2.037).

**Table 14:** *PADI4* haplotype frequencies (%) in cases and controls and risk of ESCC

Haplotypes	Case (freq)	Control (freq)	Crude OR (95% CI)	*p*
*PADI4* A_rs2477137_C_rs1886302_G_rs11203366_A_rs16825533_A_rs2240337_C_rs1635564_A_rs1635562_	63 (0.056)	65 (0.055)	0.964 [0.673∼1.383]	0.844
*PADI4* **A_rs2477137_C_rs1886302_G_rs11203366_G_rs16825533_G_rs2240337_A_rs1635564_A_rs1635562_**	**22 (0.019)**	**43 (0.036)**	**0.491 [0.290∼0.831]**	**0.007**
*PADI4* C_rs2477137_C_rs1886302_G_rs11203366_A_rs1635564_G_rs2240337_A_rs1635564_A_rs1635562_	44 (0.040)	28 (0.024)	1.599 [0.989∼2.585]	0.054
*PADI4* C_rs2477137_C_rs1886302_G_rs11203366_A_rs1635564_G_rs2240337_C_rs1635564_A_rs1635562_	56 (0.050)	47 (0.040)	1.209 [0.811∼1.803]	0.351
*PADI4* C_rs2477137_C_rs1886302_G_rs11203366_A_rs1635564_G_rs2240337_C_rs1635564_T_rs1635562_	50 (0.044)	50 (0.043)	0.985 [0.658∼1.474]	0.941
*PADI4* **C_rs2477137_T_rs1886302_A_rs11203366_A_rs1635564_G_rs2240337_A_rs1635564_A_rs1635562_**	**81 (0.073)**	**58 (0.049)**	**1.435 [1.011∼2.037]**	**0.042**
*PADI4* C_rs2477137_T_rs1886302_A_rs11203366_A_rs1635564_G_rs2240337_C_rs1635564_A_rs1635562_	285 (0.255)	288 (0.245)	0.984 [0.807∼1.201]	0.877
*PADI4* C_rs2477137_T_rs1886302_A_rs11203366_A_rs1635564_G_rs2240337_C_rs1635564_T_rs1635562_	212 (0.190)	216 (0.183)	0.981 [0.789∼1.219]	0.859
*PADI4* C_rs2477137_T_rs1886302_G_rs11203366_A_rs1635564_G_rs2240337_C_rs1635564_A_rs1635562_	60 (0.054)	63 (0.053)	0.960 [0.665∼1.385]	0.827
*PADI4* **C_rs2477137_T_rs1886302_G_rs11203366_A_rs1635564_G_rs2240337_C_rs1635564_T_rs1635562_**	**21 (0.019)**	**37 (0.031)**	**0.568 [0.330∼0.975]**	**0.038**

Haplotypes were composited by *PADI4* rs2477137, rs1886302, rs11203366, rs16825533, rs2240337, rs1635564 and rs1635562

All those frequency <0.03 were ignored in analysis

Haplotype *PADI4* **A**_rs2477137_**C**_rs1886302_**G**_rs11203366_**G**_rs16825533_**G**_rs2240337_**A**_rs1635564_**A**_rs1635562_ frequency was significantly lower in ESCC cases as compared with controls (0.019 vs. 0.036, *p*=0.007), suggesting that haplotype *PADI4* **A**_rs2477137_**C**_rs1886302_**G**_rs11203366_**G**_rs16825533_**G**_rs2240337_**A**_rs1635564_**A**_rs1635562_ genetic polymorphism may be correlated with decreased susceptibility of ESCC (OR=0.491, 95%CI: 0.290-0.831).

Haplotype *PADI4* **C**_rs2477137_**T**_rs1886302_**A**_rs11203366_**A**_rs1635564_**G**_rs2240337_**A**_rs1635564_**A**_rs1635562_ frequency was significantly higher in ESCC cases as compared with controls (0.073 vs. 0.049, *p*=0.042), suggesting that haplotype *PADI4* **C**_rs2477137_**T**_rs1886302_**A**_rs11203366_**A**_rs1635564_**G**_rs2240337_**A**_rs1635564_**A**_rs1635562_ genetic polymorphism may be correlated with increased susceptibility of ESCC (OR=1.435, 95%CI: 1.011-2.037).

Haplotype *PADI4* **C**_rs2477137_**T**_rs1886302_**G**_rs11203366_**A**_rs1635564_**G**_rs2240337_**C**_rs1635564_**T**_rs1635562_ frequency was significantly lower in ESCC cases as compared with controls (0.019 vs. 0.031, *p*=0.038), suggesting that haplotype *PADI4* **C**_rs2477137_**T**_rs1886302_**G**_rs11203366_**A**_rs1635564_**G**_rs2240337_**C**_rs1635564_**T**_rs1635562_ genetic polymorphism may be correlated with a decreased susceptibility of ESCC (OR=0.568, 95%CI: 0.330-0.975).

### Power calculation

The power calculation was performed by “Power and Sample Size Calculation” Software (http://biostat.mc.vanderbilt.edu/wiki/Main/PowerSampleSize). Based on the assumption that the type I error probability for a two sided test (α) equals 0.05, the probability of exposure in controls p0 is 0.0698 in rs2240337 in the Chinese Han population according to the NCBI project. In the current study, using ligation detection reaction method, the successful rates of genotyping all exceeded 95%. There were 1,200 alleles successfully genotyped. The ratio of control/case (m) equals 1.085, and the correlation coefficient for exposure between matched case and controls (f) is 2.058 in rs2240337. The power value is 1.000.

## DISCUSSION

In this hospital-based case-control epidemiological study in a Chinese population, we investigated whether tagging SNPs in *PADI4* were associated with risk of developing ESCC. We found that the *PADI4* rs2240337 G>A SNP was significantly associated with decreased risk of ESCC after the Bonferroni correction. *PADI4* rs11203366, rs1886302, rs1635562, rs1635564 and rs2477137 polymorphisms were implicated with altered susceptibility of ESCC according to age, gender, smoking and alcohol drinking stratification analyses.

Recently, *PADI4* has emerged as a novel transcriptional corepressor [14–16]. This enzyme catalyzes the posttranslational modification of arginine residues (to form citrulline) in histones H2A, H3, and H4 at the estrogen-regulated pS2 promoter [15–17] and at the apoptosis-related gene promoters *p21* and *OKL38* [14, 18], thereby repressing gene transcription. Additionally, the histone deaminating activity of PADI4 has been shown to downregulate the expression of numerous p53-dependent genes, including p21, PUMA, and GADD45 [14, 18]. PADI4 is overexpressed in numerous malignant cancers (e.g., breast, metastatic carcinomas, colon, bladder, lung, ovarian, and many others). In parallel, under normal circumstances, PADI4 exists as an intracellular protein, but in patients with malignant tumors, PADI4 can be detected in the plasma [16]. The PADI4 in blood increased in the presence of tumor and decreased after the tumor excision [12]. These studies bolstered the pathogenic role of PADI4 during carcinogenesis. Furthermore, expression of PADI4 was detected in esophageal cancer, but not in normal tissues. Significantly, PADI4 levels were positively correlated with the pathological classification of esophageal cancer [13].

In the present study, seven *PADI4* gene variations in Chinese population were tested and associations between these variations and outcomes in ESCC were explored. Of the seven SNPs, rs2240337 G>A was validated as an ESCC susceptibility locus, showing highly significant evidence both in heterozygote group (*p*<0.0001) and homozygote group (*p*<0.004). A previous study in a small cohort of patients with EC (83 cases and 67 controls, including ESCC and EAC) has reported that the *PADI4* rs10437048 genotype was significantly associated with decreased risk of EC, whereas rs41265997 were significantly associated with increased risk of EC [13]. In comparison with the cohort comprising ESCC and EAC in their study, we specifically focused on the relationship between ESCC and *PADI4* in a larger cohort from East China, the seemingly discrepancy with previous findings may be attributed to the distinctive genetic variants characteristics in ESCC rather than EAC. In addition, the pairwise LD tagging approach for tagging SNPs selection in this study could possibly miss some SNPs in LD with rs2240337which were also susceptibility loci for ESCC. Notably, the frequencies of genetic polymorphisms vary drastically among different ethnic cohorts.

Rs2240337 is located in the intron region of *PADI4* gene. The functions of SNPs in intron regions have not been fully elucidated. One study showed that rs2240337 could influence the mRNA stability or maturation *in vitro* [19], while the association between this SNP and rheumatoid arthritis severity has also been reported [20]. As the sample size was limited in our study, the correlation between rs2240337 and the pathologic character of ESCC was not evident, further investigation is desirable to demonstrate the functional relevance of rs2240337 polymorphism in ESCC.

Smoking and alcohol drinking have emerged as widely acknowledged risk factors of ESCC. This notion was in line with our finding, although *PADI4* rs11203366, rs1886302, rs1635562, rs1635564, rs16825533 and rs2477137 were not associated with the susceptibility to ESCC, smoking significantly increased ESCC risk in *PADI4* rs11203366 AG/AA, rs1886302 CC/CT, rs1635562 AT, rs1635564 CA and rs2477137 AC genotype, while alcohol drinking increased ESCC risk in *PADI4* rs11203366 AG, rs1635562 AT, rs1635564 CA, rs2477137 AC, rs1886302 CT genotype. Interestingly, despite the fact that rs2240337 SNP was associated with decreased risk of ESCC, smoking increased ESCC risk in *PADI4* rs2240337 AG genotype as compared with non-smokers. Our findings exemplified the significance of the environment and genetic risk factors interact and both contribute to the carcinogenesis. Our study showed the haplotype *PADI4* **A**_rs2477137_**C**_rs1886302_**G**_rs11203366_**G**_rs16825533_**G**_rs2240337_**A**_rs1635564_**A**_rs1635562_ and *PADI4* **C**_rs2477137_**T**_rs1886302_**G**_rs11203366_**A**_rs1635564_**G**_rs2240337_**C**_rs1635564_**T**_rs1635562_ genetic polymorphism may be correlated with decreased susceptibility to ESCC, while haplotype *PADI4* **C**_rs2477137_**T**_rs1886302_**A**_rs11203366_**A**_rs1635564_**G**_rs2240337_**A**_rs1635564_**A**_rs1635562_ genetic polymorphism may be correlated with increased susceptibility of ESCC, which indicated that single locus polymorphism might not significantly modify the susceptibility to cancer, the chain effect lying in different loci leads to a more profound impact on the risk of cancer.

Our study provides the evidence that polymorphism of *PIDA4* rs2240337 G>A is associated with the altered susceptibility of ESCC. We acknowledge there are several limitations in this study. First of all, the study subjects were all recruited from several local medical centers within same area, which might not completely represent the general Chinese population, especially when diverse regional environmental factors existed. Secondly, the detailed information regarding cancer metastasis and survival were not provided as the follow-up study is still ongoing, which hindered analyses of the impact of these SNP polymorphisms on ESCC progression and prognosis. Further studies with more loci and large sample size are warranted to elucidate the effect of *PADI4* SNPs on ESCC risk. Last but not least, refrained by the limited technical support, we have not evaluated the biological function of the SNP polymorphism in the carcinogenesis of ESCC in the current study. As rs2240337 is located in the intron region of *PADI4* gene, therefore overexpression of wild type and mutant type *PADI4* coding sequence does not work. We speculate that rs2240337 may cause an alternative RNA splicing on *PADI4* mRNA, thereby regulating the PADI4 protein function. Further studies using an rs2240337 G>A mutation cell or mouse model are needed to clarify the mutant PADI4 function.

## MATERIALS AND METHODS

### Ethical approval of the study protocol

We have complied with the World Medical Association Declaration of Helsinki regarding ethical conduct of research involving human subjects and/or animals. The Review Board of Jiangsu University (Zhenjiang, China) approved this hospital-based case-control study. To be included in the study, all subjects provided written informed consent.

### Patients and controls

Between October 2008 and June 2013, 629 subjects with ESCC were consecutively recruited from the Affiliated People’s Hospital of Jiangsu University and Affiliated Hospital of Jiangsu University (Zhenjiang, China). All cases of ESCC were diagnosed pathologically. The exclusion criteria were patients who previously had: cancer; any metastasized cancer; radiotherapy or chemotherapy. The 686 controls were patients without cancer and were matched to the cases with regard to age (±5 years) and sex. Most of the controls were admitted to the hospitals for the treatment of trauma. They were recruited from the two hospitals mentioned above during the same time period.

Trained interviewers, using a pre-tested questionnaire, questioned each subject personally to obtain information on demographic data (e.g., age, sex) and related risk factors (including tobacco smoking and alcohol consumption). After the interview, 2mL of venous blood was collected from each subject. Individuals who smoked one cigarette per day for >1 year were defined as “smokers”. Subjects who consumed more than three alcoholic drinks a week for >6 months were considered to be “alcohol drinkers”.

### Isolation of DNA, SNPs selection and genotyping by ligation detection reaction

Blood samples were collected from patients using vacutainers and transferred to tubes lined with ethylenediamine tetra-acetic acid (EDTA). Genomic DNA was isolated from whole blood with the QIAamp DNA Blood Mini Kit (Qiagen, Berlin, Germany) as described [21].

To find tagging SNPs, we used a block-based tagging strategy using Haploview 4.2 software, according to the HapMap database (http://www.hapmap.org/, phase II Nov08, on NCBI B36 assembly, dbSNP b126; population: Chinese Han population). Seven *PADI4* tagging SNPs were selected on the basis of Hardy-Weinberg equilibrium (HWE) *p* ≥ 0.05, call rate ≥ 95% and minor allele frequency ≥ 0.05. The samples were genotyped using the ligation detection reaction (LDR) method, with technical support from the Shanghai Biowing Applied Biotechnology Company [22]. For quality control, repeated analyses were done for 110 (11.73%) randomly selected samples with high DNA quality.

### Statistical analyses

Differences in the distributions of demographic characteristics, selected variables, genotypes of the *PADI4* variants, and the correlation between genotyping and pathologic state were evaluated using the *χ*^2^ test. The associations between the seven SNPs and risk of ESCC were estimated by computing the odds ratios (ORs) and their 95% confidence intervals (CIs) using logistic regression analyses for crude ORs and adjusted ORs when adjusting for age, sex, smoking and drinking status. The HWE was tested by a goodness-of-fit *χ*^2^ test to compare the observed genotype frequencies to the expected frequencies among the control subjects. The Bonferroni correction procedure was applied because of the number of comparisons. As multiple hypotheses are tested, the chance of a rare event increases, and the likelihood of incorrectly rejecting a null hypothesis (type I error) increases, the Bonferroni correction was therefore performed. All statistical analyses were performed with SPSS 23.0 Statistical Package (SPSS Inc., Chicago, IL).

## Abbreviations

PADI4: peptidylarginine deiminase type 4
ESCC: esophageal squamous cell carcinoma
EC: esophageal cancer
SNP: Single nucleotide polymorphism
LD: linkage disequilibrium
OR: odds ratio
CI: confidential interval
